# CSF Pulsation Artifacts on ADC Maps Obtained with Readout-segmented EPI

**DOI:** 10.2463/mrms.mp.2016-0031

**Published:** 2016-07-13

**Authors:** Toshio Ohashi, Shinji Naganawa, Mai Kanou, Mitsuru Ikeda

**Affiliations:** 1 Department of Radiology, Kamiiida Daiichi General Hospital; 2 Department of Radiology, Nagoya University Graduate School of Medicine 65 Tsurumai-cho, Shouwa-ku, Nagoya, Aichi 466-8550, Japan; 3 Department of Radiological and Medical Laboratory Sciences, Nagoya University Graduate School of Medicine

**Keywords:** magnetic resonance imaging, diffusion-weighted image, readout-segmented EPI

## Abstract

**Purpose:**

Diffusion-weighted imaging (DWI) using readout-segmented EPI (rs-EPI) can minimize distortion and blurring artifacts; however, we sometimes encounter cerebrospinal fluid (CSF) pulsation artifacts on apparent diffusion coefficient (ADC) maps, especially when the number of readout segments (NRS) is increased. The purpose of this study was to evaluate the effect of the NRS on the CSF pulsation artifacts in the ADC maps of healthy volunteers.

**Methods:**

In 10 healthy volunteers, we obtained DWI from rs-EPI with a *b*-value of 0 and 1000 s/mm^2^. The NRS was set to 5, 7, or 9. An ADC map was generated from the trace image and the *b* = 0 image. Each scan was performed twice. A circular region of interest (ROI) was drawn in the pons and bilateral thalami. The standard deviation (SD) of the ROI was measured to assess the artifacts. Bilateral SD values were averaged for the ROIs in the thalami. The SD values from two successive scans of each NRS were averaged for the pons and thalami, respectively. For the qualitative analysis, the CSF pulsation artifacts on each ADC map were graded by two observers independently as 0, no artifact; 1, mild artifact; 2, moderate artifact; or 3, severe artifact.

**Results:**

In the quantitative analysis, the SD values tend to increase with the increasing of NRS in both thalami and pons; however, the difference in the SD values from each NRS did not reach a statistically significant level. In the qualitative analysis, there was a statistically significant difference in the scores between 5 and 9 segments and between 7 and 9 segments with both the observers, respectively (*P* < 0.05).

**Conclusion:**

The CSF pulsation artifacts on ADC maps obtained with rs-EPI are affected by the NRS.

## Introduction

Diffusion-weighted imaging (DWI) obtained from readout-segmented echo-planar imaging (rs-EPI) has been utilized clinically to minimize distortion and blurring artifacts that are seen frequently on single-shot EPI (ss-EPI).^1–4^ In DWI obtained from rs-EPI, the *k*-space trajectory for each shot is similar to that from ss-EPI, but instead of traversing all *kx* points, only a readout segment is sampled. The short *kx* range makes it possible to use shorter echo-spacing, thereby reducing susceptibility artifacts. The navigator signal acquired using the second spin echo samples the central *kx* segment. Because the data points from each shot are contiguous in *k-*space, the Nyquist sampling condition is fulfilled, making it possible to use the navigational data to apply a two-dimensional (2D) phase correction in the image domain. This allows the correction of the nonlinear phase errors that are the characteristic of the non-rigid deformations of the brain which are related to the cardiac cycle.^3,5^

**Fig 1.**
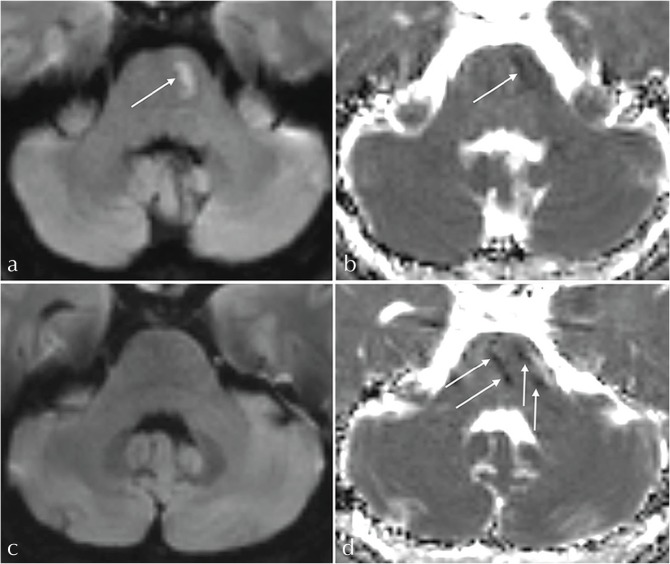
Examples of a true positive (**a, b**) and a false positive case (**c, d**). (**a**) Subacute infarction shows a high signal intensity (arrow) in the left side of the pons on the diffusion-weighted image (DWI) by readout-segmented EPI (rs-EPI) (b = 1000, number of readout segments [NRS] = 5). (**b**) On the apparent diffusion coefficient (ADC) map, this infarction shows slightly decreased values (arrow). (**c**) In another patient, there is no abnormal signal in the pons on the DWI obtained with the same parameters as (**a**). (**d**) Cerebrospinal fluid (CSF) pulsation artifacts are seen on the ADC map (arrows).

We have employed DWI obtained with rs-EPI as a routine brain imaging protocol to obtain less distorted images with a reduced number of susceptibility artifacts in the area near the skull base compared to DWI with ss-EPI. However, we occasionally noticed cerebrospinal fluid (CSF) pulsation artifacts in the thalami and pons in the readout direction on apparent diffusion coefficient (ADC) maps derived from rs-EPI, especially when a higher number of readout segments (NRS) were employed. These artifacts can mimic real pathology such as an acute infarction (Fig. 1). We have not seen such apparent CSF pulsation artifacts on ADC maps obtained from ss-EPI.

The purpose of this study was to evaluate the effect of the number of segments in the readout direction of rs-EPI on the CSF pulsation artifacts of an ADC map in healthy volunteers.

## Materials and Methods

### Volunteers and MR imaging

Ten healthy volunteers (5 men, 5 women; age range, 22–44; median, 28.5 years old) were enrolled in this study.

For the DWI obtained from the rs-EPI sequence, the second echo was used for the 2D-navigator echo to compensate for the spatially dependent phase variation caused by motion during the diffusion-sensitizing gradients. This motion-induced phase variation is different from one excitation to the next. Although 2D-navigator correction is usually effective at removing the shot-to-shot nonlinear phase variation in multi-shot DWI, the correction fails for severely corrupted data sets, in which the signal voids occur in the navigator images.^3^

In this study, the reacquisition mode^3^ was set to “on” to reacquire the severely corrupted data. This mode allowed the replacement of data up to 20%.

In 10 volunteers, we obtained DWI with rs-EPI using the number of segments in the readout direction as 5, 7, or 9. Each rs-EPI scan was obtained twice. All images were obtained on a 3T magenetic resonance (MR) imaging (MAGNETOM Skyra, SIEMENS medical solutions, Erlangen, Germany) using a 32 channel-phased array head coil. The other parameters were: repetition time (TR) of 4000 ms, echo time (TE) of 64 ms, matrix size of 160 × 160, 23 cm square field-of-view, 5 mm thick with 1.5 mm gap, and *b*-factors of 0 and 1000 s/mm^2^. The details of the scan parameters are listed in Table 1. An ADC map was generated on the console from the *b =* 0 s/mm^2^ images and the *b =* 1000 s/mm^2^ trace images according to the formula given below.

ADC = −ln(*S*_1000_/*S*_0_)/1000, where *S*_1000_ is the signal intensity on the trace-weighted image acquired with *b* = 1000 s/mm^2^ and *S*_0_ is the signal intensity on the *b* = 0 s/mm^2^ image.^6^

This study was approved by the medical ethics committee of the hospital and informed consent was obtained from all volunteers.

### Quantitative analysis

**Fig 2.**
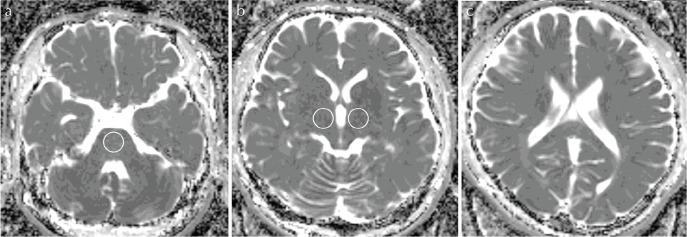
The two slice levels used for the quantitative analysis (**a**, **b**) and three slice levels used for the qualitative analysis (**a**–**c**), for the cerebrospinal fluid (CSF) artifact grading on the apparent diffusion coefficient (ADC) map. Slice in the pons (**a**), thalami (**b**), and lateral ventricle **(c)**. Examples for the circular region of interest (ROI) setting for the quantitative analysis (**a**, **b**) are also shown. The lateral ventricle level slice is used only for the qualitative analysis.

On each ADC map, one observer placed three circular regions of interests (ROIs) with a diameter of 15 mm in the bilateral thalami and the center part of the pons (Fig. 2). The ADC values and the standard deviation (SD) in the ROIs were measured. It is assumed that the SD in the ROIs would increase if there was an increase in the CSF pulsation artifacts. The SD values from both the thalami were averaged. The SD values from two successive scans at each number of segments were averaged for the pons and thalami, respectively. The mean SD value was compared between each number of segments.

### Qualitative analysis

**Fig 3.**
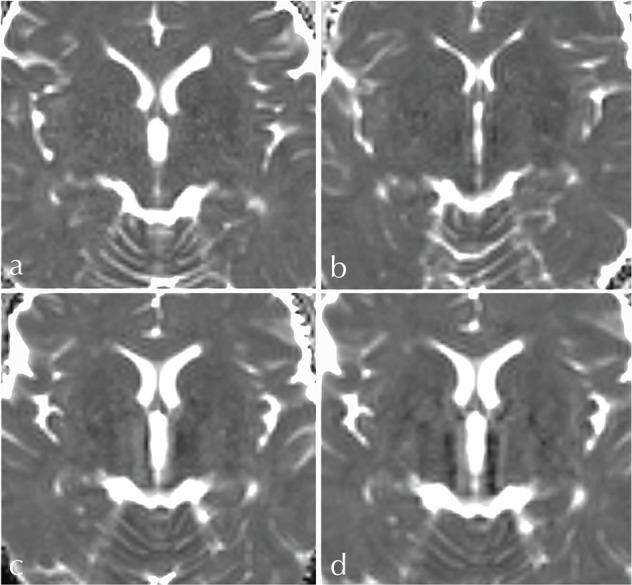
Reference examples for the qualitative artifact grading on the apparent diffusion coefficient (ADC) map. (**a**) Score 0, no artifact; (**b**) Score 1, mild artifact; (**c**) Score 2, moderate artifact; (**d**) Score 3, severe artifact

The degree of CSF pulsation artifacts on each ADC map was subjectively scored by the two observers independently as 0, no artifact; 1, mild artifact; 2, moderate artifact; or 3, severe artifact. The two observers were blinded to the scan parameters. Example images with scoring by an experienced neuroradiologist were shown to the two observers before they began scoring (Fig. 3). Images of the pons, bilateral thalami, and the body of lateral ventricles (Fig. 2) were assessed, and the maximum value of the three levels was employed. Then, the maximum value of two successive scans was selected for the subjective score value. These subjective scores from the ADC maps of 10 volunteers were compared between each NRS.

### Statistical analysis

The Friedman rank sum test was used to detect significant differences in the SD of the MR signal intensity within an ROI and in the qualitative evaluation scores for the CSF artifacts between the images obtained from 5, 7, or 9 readout segments. If the Friedman’s test was significant, pairwise comparisons were performed using a Wilcoxon-signed rank test with a Holm correction. The inter-observer agreement of the qualitative evaluation scores for 20 scan sets was evaluated with the Cohen’s kappa statistic. We used R software (version 3.2.3) for all statistical analyses, and adopted 5% as the significance level for the statistical test.

## Results

**Fig 4.**
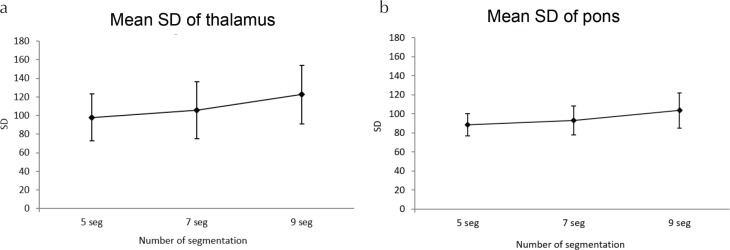
Results of the quantitative analysis in the pons and thalami. The standard deviation (SD) values of the region of interest (ROI) in the thalami (**a**) and that in the pons (**b**). Although the mean SD value with 9 readout segments in the high SD group seems to be higher than that of the 5 and 7 readout segments, the difference does not reach a statistically significant level.

Figure 4 shows the relationships between the SDs of MR signal intensity within the ROIs and the 3 readout segments for the pons and thalami. There was no statistically significant difference in the median values of the SDs between the 3 readout segments. The mean SD increased in both the thalami and the pons while the NRS increased; however, the difference did not reach a statistically significant level in either the thalami or the pons.

**Fig 5.**
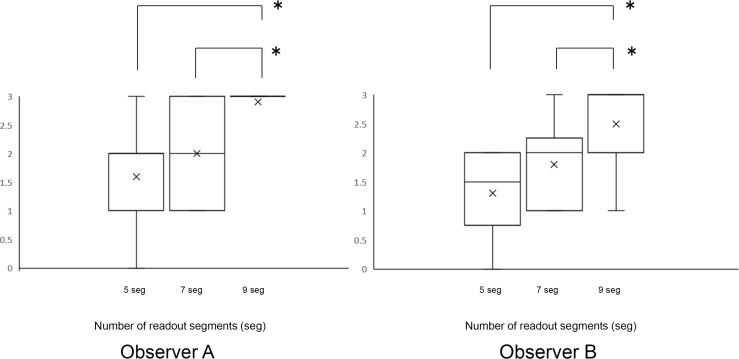
Results of the qualitative analysis by the two observers. There was a statistically significant difference in the median values of the visual evaluation scores between the different number of readout segments (NRS) in both observers **A** and **B** (*P* < 0.01). From the pairwise comparisons, there was a statistically significant difference in the median values of the scores between the 5 and 9 segments and between the 7 and 9 segments from both the observers (*P* < 0.05). *P < 0.05

Figure 5a shows the relationships between the qualitative evaluation scores of observer A and the NRS. There was a statistically significant difference in the median values of the visual evaluation scores between the 3 readout segments (*P* < 0.01). Furthermore, from the pairwise comparisons between them, there was a statistically significant difference in the median values of the scores between the 5 and 9 readout segments and between the 7 and 9 readout segments. Figure 5b shows the relationships between the qualitative evaluation scores of observer B and the NRS. There was also a statistically significant difference in the median values of the visual evaluation scores between the 3 readout segments (*P* < 0.01). Furthermore, from the pairwise comparisons between them, there was a statistically significant difference in the median values of the scores between the 5 and 9 readout segments and between the 7 and 9 readout segments.

**Fig 6.**
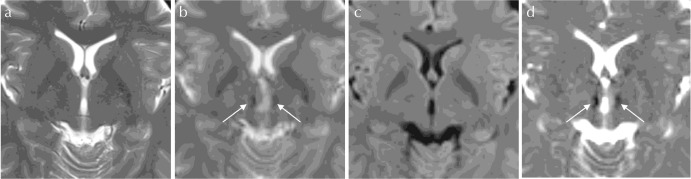
Representative images. (**a**) An axial T_2_-weighted image obtained by a two-dimensional (2D)-turbo spin echo sequence. There is no apparent cerebrospinal fluid (CSF) pulsation artifact around the third ventricle. (**b**) A corresponding slice from a T_2_-weighted readout-segmented EPI (rs-EPI) (*b* = 0 s/mm^2^) image. There are CSF pulsation artifacts in the third ventricle in the right–left (readout) direction (arrows). (**c**) A corresponding slice from a diffusion-weighted rs-EPI (*b* = 1000 s/mm^2^) image. There is no apparent CSF pulsation artifact around the third ventricle. (**d**) A corresponding slice from an apparent diffusion coefficient (ADC) map. There are severe CSF pulsation artifacts in the third ventricle in the right–left (readout) direction (arrows). A mismatch of the artifact between the images obtained at *b* = 0 s/mm^2^ and *b* = 1000 s/mm^2^ might be the cause of the artifacts on the ADC map.

The Cohen’s kappa value for the two observers’ scores was 0.426 (moderate agreement). The representative images are shown in Fig. 6.

## Discussion

The DWIs are widely used for the assessment of various neurological disorders.^7–10^ The quantitative evaluation of mean diffusivity or ADC is utilized in a variety of studies.^11^ DWI obtained with rs-EPI is strongly expected to minimize distortion and blurring and to enhance the utility of a DWI study.^1,12,13^ However, the CSF pulsation artifacts shown in this study might limit its robustness, not only in a clinical setting, but also in advanced neuroimaging research. It is important to know the cause and features of the artifacts on quantitative images.

We speculate that the cause of CSF pulsation artifacts on the ADC map obtained from rs-EPI is due to a mismatch of the pulsation artifact on the image with the *b* = 0 s/mm^2^ with that on the image with the *b* = 1000 s/mm^2^. On the image with the *b* = 0 s/mm^2^, the CSF shows as a bright signal and causes a pulsation artifact in the readout encoding direction, which is not seen on the image with *b* = 1000 s/mm^2^ (Fig. 6). With rs-EPI, each view or shot is sometimes obtained during the systolic phase and sometimes obtained during the diastolic phase. This causes a variation in the signal magnitude of flowing CSF between each shot. By Fourier transformation, this variation causes a side lobe of the object (i.e., pulsation artifact).^14^ On the other hand, CSF has a very low signal on the *b* = 1000 s/mm^2^ image, and therefore, does not produce pulsation artifact. The mismatch of the CSF pulsation artifacts on the *b* = 0 s/mm^2^ and *b* = 1000 s/mm^2^ images causes the CSF pulsation artifacts on the ADC map produced from these images. By increasing NRS, a number of discontinued or mismatched borders between the readout segments might also increase. These increased mismatched borders might also result in the image degradation.

In this study, the CSF pulsation artifacts on the ADC maps obtained from rs-EPI increased as the NRS increased from 5, 7, and 9 in the qualitative analysis. It is unknown whether the artifacts would further increase when the NRS is increased to >9. In addition to the NRS, the CSF pulsation artifact might be also affected by heart rate, arrhythmia, flow velocity of CSF, slice thickness, TR, and so on. Further detailed study is necessary to find out the relationship between the CSF pulsation artifact and these parameters.

Fractional anisotropy (FA) in diffusion tensor imaging is obtained from DWIs with motion-probing gradients (MPG) in more than six directions and a *b* = 0 image. Therefore, an FA map produced from rs-EPI data might also be susceptible to CSF pulsation artifacts similar to an ADC map.

There might be some practical solutions to minimize CSF pulsation artifacts, although further validation studies are needed. In this study, the NRS of 5 and 7 did not show a significant difference. If the degree of distortion artifact with the NRS of 5 or 7 is acceptable for the purpose of an examination, it is advised not to further increase the NRS. Images with a non-zero *b*-value for the lower *b*-value might reduce CSF pulsation artifacts by decreasing the signal of the CSF; however, the ADC value itself would change by this approach. Furthermore, we would have to acquire images with a lower *b*-value in the same number of directions as the high *b*-values images. This would increase the scan time as compared to that using a *b* = 0 s/mm^2^ image. The ECG gating might also help to reduce artifacts but it would also decrease the scan time efficiency and the variability of the R-R interval by the ECG gating might also affect the longitudinal relaxation.^3^ A 3D slab excitation would reduce the in-flow effect of the CSF, but would require a quite sophisticated pulse sequence and a longer scan time.^15^ A non-Cartesian *k*-space trajectory using the short-axis propeller (SAP)^16^ technique would be expected to scatter the pulsation artifact throughout the field-of-view. A direct comparison between the ADC maps from SAP to that from rs-EPI would be an interesting future research.

There are some limitations to this study. We scanned only a small number of young healthy volunteers. We do not know whether aged subjects with brain atrophy or with hydrocephalus would show similar results. Besides varying the NRS, we only employed fixed scan parameters such as a TR of 4000 ms, a TE of 64 ms, and a slice thickness of 5 mm. These parameters could also affect the degree of pulsation artifact.^14^ Further study with a greater variety of scan parameters is warranted. In this study, the quantitative analysis did not reach a statistically significant difference. Assessment of SD in ROI might not be as sensitive as qualitative analysis.

## Conclusion

CSF pulsation artifacts on ADC maps obtained from rs-EPI are affected by the NRS. An awareness of CSF pulsation artifacts on ADC maps from rs-EPI might be important in the field of neuroimaging research as well as for clinical neuroimaging.

## Figures and Tables

**Table 1.** Magnetic resonance (MR) pulse sequence parameters

Parameter	rs-EPI (reacquisition mode: ON)
Repetition time/echo time (ms)	4000/64
Number of slices	19
Fat saturation	CHESS
Slice thickness/gap (mm)	5.0/1.5
Field-of-view (mm)	230 × 230
Resolution (mm)	1.44 × 1.44
Matrix	160 × 160
Half Fourier	NA
Parallel imaging/accel. factor	GRAPPA/2
EPI factor	80
Band width (Hz/Px)	919, 679, 558
Number of diffusion direction	3
*b*-factor (s/mm^2^)	0, 1000
Readout direction	R–L
Number of readout segmentation	5, 7, 9
Acquisition time (min)	1:50, 2:30, 3:06

rs-EPI, readout-segmented echo-planar imaging; CHESS, chemical shift selective; GRAPPA, generalized autocalibrating partially parallel acquisition; NA, not applicable; R–L, right to left.
